# Correction: The psychological impact of the COVID-19 pandemic in Portugal: The role of personality traits and emotion regulation strategies

**DOI:** 10.1371/journal.pone.0293490

**Published:** 2023-10-23

**Authors:** Bruno Kluwe-Schiavon, Lucas De Zorzi, Joana Meireles, Jorge Leite, Henrique Sequeira, Sandra Carvalho

There are errors in the Funding section. The correct Funding statement is: SC and BKS were supported by the Portuguese Foundation for Science and Technology and the Portuguese Ministry of Science, through national funds and co-financed by FEDER through COMPETE2020 under the PT2020 Partnership Agreement (POCI-01-0145- FEDER007653) and along with the grant PTDC/PSI-ESP/ 29701/2017. JL was funded through COMPETE 2020 –PO Competitividade e Internacionalizacão/ Portugal 2020/União Europeia, FEDER (Fundos Europeus Estruturais e de Investimento–FEEI) under the number: PTDC/PSI-ESP/30280/2017. HS and LDZ are co-financed by the ESTRA International Associated Laboratory (LAI, University of Lille) and the CNRS funds to the DEEP Team of SCALab.

Funders did not play any role in the study design, data collection and analysis, decision to publish, or preparation of the manuscript.
