# Supplementary figures and images for: The psychological impact of the COVID-19 pandemic in Portugal: The role of personality traits and emotion regulation strategies
Source: PLoS One. 2022 Jun 17;17(6):e0269496. doi: 10.1371/journal.pone.0269496 (PMC9205515; doi:10.1371/journal.pone.0269496)

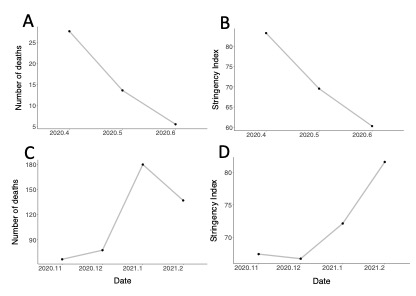

Supplement: S1 Fig — (JPG) [file pone.0269496.s001.jpg]

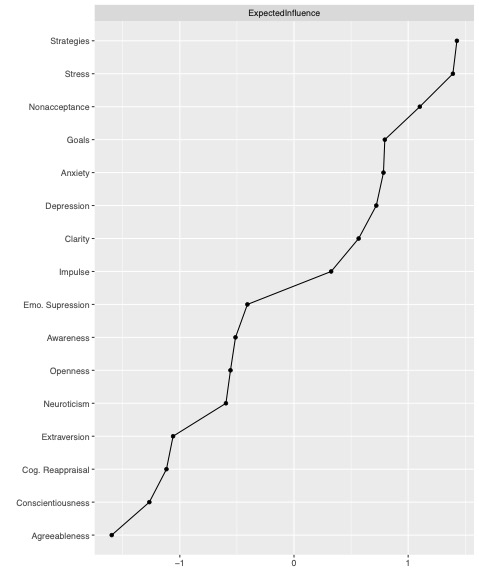

Supplement: S2 Fig — (JPG) [file pone.0269496.s002.jpg]
