# Supplementary material for: The psychological impact of the COVID-19 pandemic in Portugal: The role of personality traits and emotion regulation strategies
Source: PLoS One. 2022 Jun 17;17(6):e0269496. doi: 10.1371/journal.pone.0269496 (PMC9205515; doi:10.1371/journal.pone.0269496)
Supplement: S1 Table — (PDF) [file pone.0269496.s003.pdf]

**Table S1:** *Distribution of emotional regulation skills, personality traits and psychological symptoms at Phase I (T0) and Phase II.*

| | Phase I (T0) n=180 | Phase II n=542 | Test statistics [df] | Effect size |
|---|---|---|---|---|
| **Difficulties in emotion regulation** | | | | |
| Clarity | 6.91 (2.84) | 8.05 (3.18) | t[340.43]=-4.55 | d=-.494*** |
| Goals | 10.14 (3.33) | 10.61 (3.08) | t[287.75]=-1.69 | d=-.199 |
| Impulse | 6.18 (3.10) | 7.72 (3.51) | t[344.24]=-5.59 | d=-.603 *** |
| Strategies | 6.92 (3.66) | 8.59 (3.52) | t[296.96]=-5.36 | d=-.623 *** |
| Awareness | 6.52 (2.41) | 6.55 (2.71) | t[341.91]=-.14 | d=-.016 |
| Non-acceptance | 6.71 (3.35) | 8.20 (3.56) | t[323.44]=-5.11 | d=-.568 *** |
| **Emotional regulation strategies** | | | | |
| Cognitive reappraisal | 28.77 (6.46) | 29.28 (6.45) | t[306.09]=-.93 | d=-.106 |
| Emotional suppression | 14.76 (4.87) | 17.37 (5.62) | t[349.80]=-5.99 | d=-.641 *** |
| **Personality traits** | | | | |
| Neuroticism, mean (SD) | 26.11 (8.42) | 27.91 (7.57) | t[281.34]=-2.55 | d=-.305 * |
| Very low, n (%) | 2 (1.1) | 4 (<0) | $X^2$[1]=.22 | V=.017 |
| Low, n (%) | 8 (4.4) | 11 (2) | $X^2$[1]=3.07 | V=.065 |
| Normal, n (%) | 42 (23.3) | 82 (15.1) | $X^2$[1]=6.39 | V=.094 * |
| High, n (%) | 64 (35.6) | 219 (40.4) | $X^2$[1]=1.33 | V=.042 |
| Very high, n (%) | 64 (35.6) | 226 (41.7) | $X^2$[1]=2.12 | V=.054 |
| Extraversion, mean (SD) | 29.36 (5.80) | 28.71 (5.97) | t[314.28]=1.30 | d=.147 |
| Very low, n (%) | 7 (3.8) | 22 (4) | $X^2$[1]=.01 | V=.003 |
| Low, n (%) | 22 (12.2) | 70 (12.9) | $X^2$[1]=.05 | V=.008 |
| Normal, n (%) | 71 (39.4) | 244 (45) | $X^2$[1]=1.70 | V=.048 |
| High, n (%) | 61 (33.8) | 156 (28.7) | $X^2$[1]=1.67 | V=.048 |
| Very high, n (%) | 19 (10.5) | 50 (9.2) | $X^2$[1]=.27 | V=.019 |
| Openness, mean (SD) | 27.61 (5.33) | 27.07 (4.82) | t[282.49]=1.20 | d=.143 |
| Very low, n (%) | 7 (3.9) | 6 (1.1) | $X^2$[1]=5.91 | V=.090 * |
| Low, n (%) | 22 (12.2) | 83 (15.3) | $X^2$[1]=.96 | V=.037 |
| Normal, n (%) | 89 (49.4) | 332 (61.2) | $X^2$[1]=7.74 | V=.103 ** |

| | | | | |
|---|---|---|---|---|
| High, n (%) | 57 (31.7) | 101 (18.9) | $X^2$[1]=13.42 | V=.136 *** |
| Very high, n (%) | 5 (2.8) | 20 (3.6) | $X^2$[1]=.33 | V=.021 |
| Agreeableness, mean (SD) | 32.26 (5.78) | 29.18 (7.11) | t[372.79]=5.82 | d=.603 *** |
| Very low, n (%) | 12 (6.6) | 126 (23.2) | $X^2$[1]=24.02 | V=182 ***. |
| Low, n (%) | 35 (19.4) | 137 (25.2) | $X^2$[1]=2.53 | V=.059 |
| Normal, n (%) | 74 (41.1) | 167 (30.8) | $X^2$[1]=6.44 | V=.094 ** |
| High, n (%) | 46 (25.5) | 74 (13.6) | $X^2$[1]=13.81 | V=.138 *** |
| Very high, n (%) | 13 (7.2) | 38 (7) | $X^2$[1]<.00 | V=.003 |
| Conscientiousness, mean (SD) | 32.53 (7.51) | 30.54 (6.42) | t[271.31]=3.19 | d=.387 ** |
| Very low, n (%) | 26 (14.4) | 80 (14.7) | $X^2$[1]=.01 | V=.003 |
| Low, n (%) | 29 (16.1) | 162 (29.8) | $X^2$[1]=13.18 | V=.135 *** |
| Normal, n (%) | 81 (45) | 226 (41.7) | $X^2$[1]=.60 | V=.028 |
| High, n (%) | 31 (17.2) | 53 (9.7) | $X^2$[1]=7.28 | V=.100 ** |
| Very high, n (%) | 13 (7.2) | 21 (3.8) | $X^2$[1]=3.37 | V=.068 |
| **Psychological symptoms** | | | | |
| Depression, mean (SD) | 5.02 (4.76) | 7.64 (5.62) | t[358.19]=-6.10 | d=-.645 *** |
| Normal, n (%) | 109 (60.5) | 198 (36.5) | $X^2$[1]=31.91 | V=.210 *** |
| Mild, n (%) | 20 (11.1) | 58 (10.7) | $X^2$[1]=.02 | V=.005 |
| Moderate, n (%) | 27 (15) | 113 (20.8) | $X^2$[1]=2.95 | V=.064 |
| Severe, n (%) | 9 (5) | 75 (13.8) | $X^2$[1]=10.26 | V=.119 ** |
| Extreme, n (%) | 15 (8.3) | 98 (18) | $X^2$[1]=9.72 | V=.116 ** |
| Anxiety, mean (SD) | 3.96 (4.59) | 6.84 (5.31) | t[350.8]=-6.99 | d=-.747 *** |
| Normal, n (%) | 105 (58.3) | 174 (32.1) | $X^2$[1]=39.21 | V=.233 ** |
| Mild, n (%) | 31 (17.2) | 84 (15.5) | $X^2$[1]=.29 | V=.020 |
| Moderate, n (%) | 12 (6.6) | 58 (10.7) | $X^2$[1]=2.51 | V=.058 |
| Severe, n (%) | 10 (5.5) | 58 (10.7) | $X^2$[1]=4.19 | V=.076 * |
| Extreme, n (%) | 22 (12.2) | 168 (31) | $X^2$[1]=24.56 | V=.184 *** |
| Stress, mean (SD) | 7.15 (4.81) | 9.83 (4.97) | t[315.31]=-6.42 | d=-.723 *** |
| Normal, n (%) | 109 (60.5) | 194 (35.7) | $X^2$[1]=34.02 | V=.217 *** |
| Mild, n (%) | 17 (9.4) | 74 (13.6) | $X^2$[1]=2.17 | V=.054 |
| Moderate, n (%) | 26 (14.4) | 97 (17.9) | $X^2$[1]=1.13 | V=.039 |
| Severe, n (%) | 20 (11.1) | 128 (23.6) | $X^2$[1]=12.96 | V=.134 *** |

| | | | | |
|---|---|---|---|---|
| Extreme, n (%) | 8 (4.4) | 49 (9) | $X^2[1]=3.92$ | V=.073 * |

**Note.** * *p*<.05; ** *p*<.01; *** *p*<.001. For continuous variables Student t-test was performed and Cohen's d effect size calculated (d, 0.1 to 0.2, weak; 0.5 to 0.8, moderate; >0.8 strong). For frequency data, chi-squared test was performed. When significant findings were observed, chi-squared pairwise comparisons were done and Cramer's V effect size calculated (V, 0.2, small; 0.2 to 0.6, medium; >0.6 large).
