# Supplementary material for: The psychological impact of the COVID-19 pandemic in Portugal: The role of personality traits and emotion regulation strategies
Source: PLoS One. 2022 Jun 17;17(6):e0269496. doi: 10.1371/journal.pone.0269496 (PMC9205515; doi:10.1371/journal.pone.0269496)
Supplement: S2 Table — (PDF) [file pone.0269496.s004.pdf]

Table S2. Stepwise linear models on Phase I and Phase II differences regarding psychological symptoms.

		Psychological symptoms		
		Depression	Anxiety	Stress
**Group**	Phase II	.56	.62	**0.78 ***
**Demographics**	Age	-.02	**1.35 ****	**-.04 ***
Region	North	-	-	-
	Center	-	-	-
Marital status	Single	-	-	-1.01
	Married / Stable union	-	-	-
Educational level	≤ to 9th Grade	**1.77 ****	**1.35 ****	**1.30 ***
	High school	-.66	-	-
Work modality	Remote	-.77	**-1.25 ****	**-.89 ***
	Suspended	-1.05	**-1.75 ***	**-1.52 ***
	Retired	-	-	-
Medical history	Neurologic Disease	-	1.48	-
	Other Condition	**.88 ***	**1.10 ****	**.73 ***
**Pandemic-related factors**	Social confinement	**1.29 *****	**1.02 ****	**.79 ***
	In quarantine	**2.90 ****	**3.03 *****	**1.92 ***
	Currently/Previously positive for COVID-19	-	-	-
	Not living in a house	-	**-0.45 ***	-
	Assess to green/public spaces	-	-	-.71
	Changes in Relationships	-	.72	**1.21 ****
**Difficulties in emotion regulation**	Clarity	**.15 ***	**.15 ***	-
	Impulse	-	**.16 ***	**.35 *****
	Strategies	**.63 *****	**.34 *****	**.40 *****
	Non-acceptance	**.15 ***	.12	-
**E. regulation strategies**	Emotional suppression	**.07 ***	**.08 ****	.04
**Personality traits**				
Neuroticism	Normal ranges	**-1.45 *****	**-1.58 *****	**-1.83 *****
Openness	Very low	-		-
	Normal	-		-
Agreeableness	Very low	-		-
	Normal	-		**.66 ***
	High	-		-
	Low	-		-
Conscientiousness				
	High	-	**1.01 ***	**.98 ***
**Model**	F	45.65	29.13	29.32
	DF	698	695	695
	Adjusted R^2	.46	.40	.40

Note. Statistically significant correlates are presented in bold. β=beta (standardized coefficient) is reported. * *p*<0.05, ** *p*<0.01, *** *p*<0.001; "-", not included by the stepwise method.
