# Supplementary material for: The psychological impact of the COVID-19 pandemic in Portugal: The role of personality traits and emotion regulation strategies
Source: PLoS One. 2022 Jun 17;17(6):e0269496. doi: 10.1371/journal.pone.0269496 (PMC9205515; doi:10.1371/journal.pone.0269496)
Supplement: S1 File — (DOCX) [file pone.0269496.s005.docx]

**The Psychological Impact of the COVID-19 Pandemic in Portugal: The Role of Personality Traits and Emotion Regulation Strategies**

**Supplementary material**

**Method**

***Instrument and questionnaires***

The online questionnaire used both in Phase I and Phase II of this study comprised instruments that assessed five main areas, namely, socio-demographic information, psychological distress, personality, emotion regulation, and effects of COVID-19 pandemic.

*Socio-demographic information*

The socio-demographic questionnaire was developed for purpose of this study and has as primary goal the demographic, medical and academic/professional characterization of the participants. It contains 15 items of direct and multiple-choice answers that gather information regarding age, gender, region of residency, marital status, educational level, professional activity, changes in work/study regime, number of family members, number of people directly dependent on the respondents, and medical history. It takes approximately 3 minutes to complete.

*Psychological distress*

Psychological distress was assessed using the Depression, Anxiety, and Stress Scale (DASS-21) (Pais-Ribeiro et al., 2004) which is subdivided in three groups of symptoms, namely, depression (α = 0.85), anxiety (α = 0.74), and stress (α = 0.81). Each group is comprised of 7 items that describe an emotionally negative symptom (e.g., “I found it difficult to relax”) that are rated according to the degree of appliance to the respondent, ranging from 0 (not applied at all) to 3 (applied to me most of the time). The final score for each scale can range between 0 to 21, and further categorized into normal depression (0 – 4), anxiety (0 – 3), and stress (0 – 7) symptomatology to extremely severe levels of depression (equal or superior to 14), anxiety (equal or superior to 10), and stress (equal or superior to 17). The sum of all three domains gives a general measure of psychological distress. The completeness of the total scale takes only 2 minutes.

*Personality traits*

Personality traits were evaluated using the NEO-FFI (Magalhães et al., 2014) which characterizes the cognitions, behaviors, and emotions of the participants regarding five personality dimensions, namely, neuroticism (α = 0.99), extraversion (α = 0.78), openness (α = 0.76), agreeableness (α = 0.86), and conscientiousness (α = 0.90). The instrument has 60 items rated in a 5-point Likert scale, ranging from 0 (strongly disagree) to 4 (strongly agree), and takes approximately 15 minutes to complete. The scores obtain in each personality trait can range from 0 to 48 with higher scores representing a greater presence of the trait in a person’s personality and lower scores indicate the opposite (e.g., scoring 11 in neuroticism represents a person with low neuroticism (or high emotional stability), while scoring 22 represents a person with high neuroticism (or low emotional stability).

*Emotional regulation*

To evaluate emotion regulation both the Difficulties of Emotion Regulation Scale (DERS-18) (Coutinho et al., 2010) and the Emotion Regulation Questionnaires (ERQ) (Vaz et al., 2014) were employed. The DERS-18 is a 18-items measure that assesses levels of emotion dysregulation according to six dimensions (each with an α > 0.75.), awareness (i.e., lack of understanding of one’s own emotions), clarity (i.e., difficulty to clarify the nature of the emotion experienced), non-acceptance (i.e., difficulties on accepting one’s emotional state), strategy (i.e., absence of effective emotion regulation strategies), goals (i.e., inability to engage in goal-directed activities while experiencing negative emotions), and impulse (i.e., incapability to manage impulses during overwhelming experience of emotions). Items are scored on a 5-point scale with higher scores representing worst adjustment. This scale can be interpreted using each dimension or the sum of all dimensions that gives a general measure of emotion dysregulation. This measure takes 3-5 minutes to fill.

The ERQ is a 10- item scale that measures individual differences in the dispositional use of two emotion regulation strategies: cognitive reappraisal (α = 0.76) and emotional suppression (α = 0.65). This 8-point Likert scale, ranging from 1 (totally disagree) to 8 (totally agree) takes only 2 minutes to complete. Example items for cognitive reappraisal and emotional suppression include: “I control my emotions by changing the way I think about the situation I’m in”, and “I control my emotions by not expressing them”, respectively. Scores can range between 6 to 48 for cognitive reappraisal and 4 to 32 for emotional suppression with higher scores representing higher disposition to use the relative emotional regulation strategy.

*Lifestyle*

To investigate how the pandemic was affecting the participants, a 26-item questionnaire was used. The questionnaire to evaluate lifestyle during COVID-19 evaluated participants contact with the virus, engagement with restrictive measures, housing characteristics, and interpersonal relationships. More precisely, participants were enquired regarding current or previous infection with COVID-19, following of voluntary/mandatory social confinement, undertaking of quarantine, housing type (e.g., house), access to outdoor spaces (e.g., public spaces near residential area), changes in relationship patterns compared to pre-pandemic period. It included direct and multiple-choice questions with an expected completion time of 5 to 7 minutes.

| **Figure S1.** Absolute number of deaths and stringency index at Phase I and II. |
| --- |
|  |
| **Note**. Data retrieved from the World Bank Data available at the R package “COVID19” (Guidotti & Ardia, 2020). |

***Regularized partial correlation***

As mentioned in the text, potentially redundant nodes among our variables were checked using a data-driven method before computing the networks. To do so, we first confirmed that our correlation matrix was positive definite (i.e., nodes are not linear combinations of other nodes) and, next, we explored nodes that are highly intercorrelated (r>0.50) and that exhibit highly similar patterns of correlations with the remaining nodes in the network, i.e., >75% of correlations with other variables did not significantly differ for a given pair. Functionally indistinguishable pairs of nodes were, therefore, identified by comparing dependent correlations (Hittner et al., 2003) using the R package “networktools” (Jones, 2018). There were no apparent redundant variables in the present dataset.

**Results**

***Distribution of psychological distress and personality traits at T0***

As shown in **Table S1**, only 17% of the participants reported severe to extreme levels of anxiety, while 15% and 13% of the participants reported severe to extreme levels of stress and depression, respectively. To further characterize the sample, personality traits were categorized into groups according to NEO-FFI scoring. Much of the sample shows high to very high levels of neuroticism (71%), relatively low levels of agreeableness (41%), low to acceptable levels of conscientiousness (45%), acceptable to high levels of extraversion (70%), and acceptable levels of openness (49%).

***Distribution of psychological distress and personality traits at Phase II***

As shown in **Table S1**, moderate to extreme depression, anxiety and stress symptoms were reported by 52%, 52%, and 50% of the respondents, respectively. To further characterize the sample, personality traits were categorized into groups according to NEO-FFI scoring. Much of the sample showed high to very high levels of neuroticism (82%), relatively low levels of agreeableness (48%), low to acceptable levels of conscientiousness (71%), acceptable to high levels of extraversion (73%), and acceptable levels of openness (61%).

Table S1: *Distribution of emotional regulation skills, personality traits and psychological symptoms at Phase I (T0) and Phase II.*

|  | Phase I (T0)  n=180 | Phase II  n=542 | Test statistics [df] | Effect size |
| --- | --- | --- | --- | --- |
| **Difficulties in emotion regulation** |  |  |  |  |
| Clarity | 6.91 (2.84) | 8.05 (3.18) | t[340.43]=-4.55 | d=-.494*** |
| Goals | 10.14 (3.33) | 10.61 (3.08) | t[287.75]=-1.69 | d=-.199 |
| Impulse | 6.18 (3.10) | 7.72 (3.51) | t[344.24]=-5.59 | d=-.603 *** |
| Strategies | 6.92 (3.66) | 8.59 (3.52) | t[296.96]=-5.36 | d=-.623 *** |
| Awareness | 6.52 (2.41) | 6.55 (2.71) | t[341.91]=-.14 | d=-.016 |
| Non-acceptance | 6.71 (3.35) | 8.20 (3.56) | t[323.44]=-5.11 | d=-.568 *** |
| **Emotional regulation strategies** |  |  |  |  |
| Cognitive reappraisal | 28.77 (6.46) | 29.28 (6.45) | t[306.09]=-.93 | d=-.106 |
| Emotional suppression | 14.76 (4.87) | 17.37 (5.62) | t[349.80]=-5.99 | d=-.641 *** |
| **Personality traits** |  |  |  |  |
| Neuroticism, mean (SD) | 26.11 (8.42) | 27.91 (7.57) | t[281.34]=-2.55 | d=-.305 * |
| Very low, n (%) | 2 (1.1) | 4 (<0) | X^2^[1]=.22 | V=.017 |
| Low, n (%) | 8 (4.4) | 11 (2) | X^2^[1]=3.07 | V=.065 |
| Normal, n (%) | 42 (23.3) | 82 (15.1) | X^2^[1]=6.39 | V=.094 * |
| High, n (%) | 64 (35.6) | 219 (40.4) | X^2^[1]=1.33 | V=.042 |
| Very high, n (%) | 64 (35.6) | 226 (41.7) | X^2^[1]=2.12 | V=.054 |
| Extraversion, mean (SD) | 29.36 (5.80) | 28.71 (5.97) | t[314.28]=1.30 | d=.147 |
| Very low, n (%) | 7 (3.8) | 22 (4) | X^2^[1]=.01 | V=.003 |
| Low, n (%) | 22 (12.2) | 70 (12.9) | X^2^[1]=.05 | V=.008 |
| Normal, n (%) | 71 (39.4) | 244 (45) | X^2^[1]=1.70 | V=.048 |
| High, n (%) | 61 (33.8) | 156 (28.7) | X^2^[1]=1.67 | V=.048 |
| Very high, n (%) | 19 (10.5) | 50 (9.2) | X^2^[1]=.27 | V=.019 |
| Openness, mean (SD) | 27.61 (5.33) | 27.07 (4.82) | t[282.49]=1.20 | d=.143 |
| Very low, n (%) | 7 (3.9) | 6 (1.1) | X^2^[1]=5.91 | V=.090 * |
| Low, n (%) | 22 (12.2) | 83 (15.3) | X^2^[1]=.96 | V=.037 |
| Normal, n (%) | 89 (49.4) | 332 (61.2) | X^2^[1]=7.74 | V=.103 ** |
| High, n (%) | 57 (31.7) | 101 (18.9) | X^2^[1]=13.42 | V=.136 *** |
| Very high, n (%) | 5 (2.8) | 20 (3.6) | X^2^[1]=.33 | V=.021 |
| Agreeableness, mean (SD) | 32.26 (5.78) | 29.18 (7.11) | t[372.79]=5.82 | d=.603 *** |
| Very low, n (%) | 12 (6.6) | 126 (23.2) | X^2^[1]=24.02 | V=182 ***. |
| Low, n (%) | 35 (19.4) | 137 (25.2) | X^2^[1]=2.53 | V=.059 |
| Normal, n (%) | 74 (41.1) | 167 (30.8) | X^2^[1]=6.44 | V=.094 ** |
| High, n (%) | 46 (25.5) | 74 (13.6) | X^2^[1]=13.81 | V=.138 *** |
| Very high, n (%) | 13 (7.2) | 38 (7) | X^2^[1]<.00 | V=.003 |
| Conscientiousness, mean (SD) | 32.53 (7.51) | 30.54 (6.42) | t[271.31]=3.19 | d=.387 ** |
| Very low, n (%) | 26 (14.4) | 80 (14.7) | X^2^[1]=.01 | V=.003 |
| Low, n (%) | 29 (16.1) | 162 (29.8) | X^2^[1]=13.18 | V=.135 *** |
| Normal, n (%) | 81 (45) | 226 (41.7) | X^2^[1]=.60 | V=.028 |
| High, n (%) | 31 (17.2) | 53 (9.7) | X^2^[1]=7.28 | V=.100 ** |
| Very high, n (%) | 13 (7.2) | 21 (3.8) | X^2^[1]=3.37 | V=.068 |
| **Psychological symptoms** |  |  |  |  |
| Depression, mean (SD) | 5.02 (4.76) | 7.64 (5.62) | t[358.19]=-6.10 | d=-.645 *** |
| Normal, n (%) | 109 (60.5) | 198 (36.5) | X^2^[1]=31.91 | V=.210 *** |
| Mild, n (%) | 20 (11.1) | 58 (10.7) | X^2^[1]=.02 | V=.005 |
| Moderate, n (%) | 27 (15) | 113 (20.8) | X^2^[1]=2.95 | V=.064 |
| Severe, n (%) | 9 (5) | 75 (13.8) | X^2^[1]=10.26 | V=.119 ** |
| Extreme, n (%) | 15 (8.3) | 98 (18) | X^2^[1]=9.72 | V=.116 ** |
| Anxiety, mean (SD) | 3.96 (4.59) | 6.84 (5.31) | t[350.8]=-6.99 | d=-.747 *** |
| Normal, n (%) | 105 (58.3) | 174 (32.1) | X^2^[1]=39.21 | V=.233 ** |
| Mild, n (%) | 31 (17.2) | 84 (15.5) | X^2^[1]=.29 | V=.020 |
| Moderate, n (%) | 12 (6.6) | 58 (10.7) | X^2^[1]=2.51 | V=.058 |
| Severe, n (%) | 10 (5.5) | 58 (10.7) | X^2^[1]=4.19 | V=.076 * |
| Extreme, n (%) | 22 (12.2) | 168 (31) | X^2^[1]=24.56 | V=.184 *** |
| Stress, mean (SD) | 7.15 (4.81) | 9.83 (4.97) | t[315.31]=-6.42 | d=-.723 *** |
| Normal, n (%) | 109 (60.5) | 194 (35.7) | X^2^[1]=34.02 | V=.217 *** |
| Mild, n (%) | 17 (9.4) | 74 (13.6) | X^2^[1]=2.17 | V=.054 |
| Moderate, n (%) | 26 (14.4) | 97 (17.9) | X^2^[1]=1.13 | V=.039 |
| Severe, n (%) | 20 (11.1) | 128 (23.6) | X^2^[1]=12.96 | V=.134 *** |
| Extreme, n (%) | 8 (4.4) | 49 (9) | X^2^[1]=3.92 | V=.073 * |
| **Note.** * *p*<.05; ** *p*<.01; *** *p*<.001. For continuous variables Student t-test was performed and Cohen’s d effect size calculated (d, 0.1 to 0.2, weak; 0.5 to 0.8, moderate; >0.8 strong). For frequency data, chi-squared test was performed. When significant findings were observed, chi-squared pairwise comparisons were done and Cramer's V effect size calculated (V, 0.2, small; 0.2 to 0.6, medium; >0.6 large). | | | | |

| **Table S2.** Stepwise linear models on Phase I and Phase II differences regarding psychological symptoms. | | | | |
| --- | --- | --- | --- | --- |
|  |  | Psychological symptoms | | |
|  |  | Depression | Anxiety | Stress |
| **Group** | Phase II | .56 | .62 | **0.78 *** |
| **Demographics** | Age | -.02 | **1.35 **** | **-.04 *** |
| Region | North | - | - | - |
|  | Center | - | - | - |
| Marital status | Single | - | - | -1.01 |
|  | Married / Stable union | - | - | - |
| Educational level | < to 9^th^ Grade | **1.77 **** | **1.35 **** | **1.30 *** |
|  | High school | -.66 | - | - |
| Work modality | Remote | -.77 | **-1.25 **** | **-.89 *** |
|  | Suspended | -1.05 | **-1.75 *** | **-1.52 *** |
|  | Retired | - | - | - |
| Medical history | Neurologic Disease | - | 1.48 | - |
|  | Other Condition | **.88 *** | **1.10 **** | **.73 *** |
| **Pandemic-related** | Social confinement | **1.29 ***** | **1.02 **** | **.79 *** |
| **factors** | In quarantine | **2.90 **** | **3.03 ***** | **1.92 *** |
|  | Currently/Previously positive for COVID-19 | - | - | - |
|  | Not living in a house | - | **-0.45 *** | - |
|  | Assess to green/public spaces | - | - | -.71 |
|  | Changes in Relationships | - | .72 | **1.21 **** |
| **Difficulties in emotion** | Clarity | **.15 *** | **.15 *** | - |
| **regulation** | Impulse | - | **.16 *** | **.35 ***** |
|  | Strategies | **.63 ***** | **.34 ***** | **.40 ***** |
|  | Non-acceptance | **.15 *** | .12 | - |
| **E. regulation strategies** | Emotional suppression | **.07 *** | **.08 **** | .04 |
| **Personality traits** |  |  |  |  |
| Neuroticism | Normal ranges | **-1.45 ***** | **-1.58 ***** | **-1.83 ***** |
| Openness | Very low | - |  | - |
|  | Normal | - |  | - |
| Agreeableness | Very low | - |  | - |
|  | Normal | - |  | **.66 *** |
|  | High | - |  | - |
| Conscientiousness | Low | - |  | - |
|  | High | - | **1.01 *** | **.98 *** |
| **Model** | F | 45.65 | 29.13 | 29.32 |
|  | DF | 698 | 695 | 695 |
|  | Adjusted R^2^ | .46 | .40 | .40 |
| **Note.** Statistically significant correlates are presented in bold. β=beta (standardized coefficient) is reported. * *p*<0.05, ** *p*<0.01, *** *p*<0.001; “-”, not included by the stepwise method. | | | | |

| **Figure S2.** Centrality indexes, strength and expected influence. |
| --- |
|  |
| **Note.** Higher expected influence values indicate greater centrality and thus greater importance in the network. The plot depicts the normalized (z-scored) expected influence centrality values for each node. |

**References**

Coutinho, J., Ribeiro, E., Ferreirinha, R., & Dias, P. (2010). Versão portuguesa da escala de dificuldades de regulação emocional e sua relação com sintomas psicopatológicos. . *Archives of Clinical Psychiatry, 37*(4), 6. <https://doi.org/https://doi.org/10.1590/s0101-60832010000400001>

Guidotti, E., & Ardia, D. (2020). *COVID-19 Data Hub*. <https://cran.r-project.org/web/packages/COVID19/COVID19.pdf>

Hittner, J., May, K., & Silver, N. (2003). A Monte Carlo evaluation of tests for comparing dependent correlations. *The Journal of General Psychology, 130*, 19. <https://doi.org/> <https://doi.org/10.1080/00221300309601282>.

Jones, P. J. (2018). Networktools: Tools for identifying important nodes in networks. *R Package Version,, 1*(2).

Magalhães, E., Salgueira, A., Gonzalez, A., Costa, J., Costa, M., Costa, P., & Lima, M. (2014). NEO-FFI: Psychometric properties of a short personality inventory in Portuguese context. *Psicologia: Reflexão e Crítica, 27*(4), 657. <https://doi.org/https://doi.org/10.1590/1678-7153.201427405>

Pais-Ribeiro, J., Honrado, A., & Leal, I. (2004). Contribuicao para o estudo da Adaptacao Portuguesa das Escalas de Ansiedade, Depressco e Stress (EADS) de 21 itens de Lovibond e Lovibond. *Psicologia, Saúde & Doencas, 5*, 10. <http://hdl.handle.net/10400.12/1058>

Vaz, F., Martins, C., & Martins, E. (2014). Diferenciação emocional e regulação emocional em adultos portugueses. *Psicologia, 22*(2). <https://doi.org/>. <https://doi.org/10.17575/rpsicol.v22i2.350>
